# Teaching population health in general practice: developing mindset through continuity, community, and data

**DOI:** 10.3389/fmed.2025.1641310

**Published:** 2025-08-29

**Authors:** Waseem Jerjes, Azeem Majeed

**Affiliations:** Department of Primary Care and Public Health, Faculty of Medicine, Imperial College London, London, United Kingdom

**Keywords:** population health, general practice, interprofessional education, continuity of care, quality improvement

## Introduction—the expanding mandate of primary care

GPs are increasingly called upon to look beyond individual consultations and address people's health more holistically (1). As of March 2025, GP practices in England were responsible for ~63.8 million registered patients, reflecting a significant increase over recent years (2). With health systems everywhere moving toward value-based care, prevention, and health equity, GPs are spearheading initiatives to tackle rising chronic disease rates, unmet social need, and system-level inequalities (3). This shift in clinical practice is reflected by an intensified focus in medical education on population health—not just a collection of technical skills or measures, but an overarching strategy for enhancing outcomes among specified patient groups.

Population health refers to the outcomes of a defined group of individuals—including the distribution of those outcomes—and the factors that influence them, such as social, economic, and environmental determinants. In general practice, this means taking responsibility not just for the person in the room, but for an entire registered list of patients. It involves thinking systemically, identifying who is missing from care, and working to address barriers that prevent equitable access or health outcomes. Simply put, it asks GPs to look beyond individual episodes—to discern patterns, understand context, and act for the whole. Population health has been succinctly defined in US literature as the health outcomes of a group of individuals, including the distribution of such outcomes within the group (4).

Yet, adopting this wider responsibility significantly increases the emotional and cognitive demands placed upon general practitioners. Without adequate mentoring, supportive teamwork, and realistic expectations, the complexity and sheer scope of primary health care can easily result in moral fatigue or professional overwhelm. Effective training must therefore equip general practitioners with the resilience and interpersonal skills necessary to manage these challenges sustainably.

Work to teach population health emphasizes teaching about clinical dashboards, disease registers, and measures of performance. These are important tools, but inadequate by themself (5). At their best, population health practice is about more than management of data—it's about a different way of thinking and working. GPs need to be educated to understand variation, observe what is not seen, and accept responsibility for communities, not just for individuals. Foundational didactic education in epidemiology, biostatistics, and the social determinants of health (SDoH) provides trainees with necessary analytical skills to interpret population-level data. Incorporating structured educational modules covering these foundational areas is essential to develop competency in population health among medical trainees (6, 7).

To equip GPs to embrace population health, training has to get beyond mere technical competency to developing a mindset of stewardship of populations. Using the UK model of GP training and applicable educational literature, we discuss how continuity, community involvement, and reflective learning add to developing practitioners suited to leading population-driven care.

## What does it mean to think in populations? Beyond technical competence

Population health is brought into curricula through tools such as dashboards, disease registers, and performance measures (8). Tools are of great worth, particularly within systems working to optimize care at scale. Tools, though, don't educate physicians how to think about populations. What is missing is often an emphasis upon developing a mindset which enables GPs to shift from executing tasks to reflective stewardship over health within a specified group of patients.

It involves a shift of direction (9). Rather than concentrating primarily on the patient before them, clinicians start to pose alternate sorts of questions: what are the patterns I am seeing in my patients? Which patients are not booking appointments? What external influences outside the consultation room could be influencing these results? This type of questioning comes from a more systemic model of care and requires not just an awareness of presence, but, equally, of absence and difference.

Central to such an attitude is an ability to think of care delivered through an intricate web of interacting clinical, social, and institutional forces. Notably, practices in the most deprived areas have, on average, 300 (14.4%) more patients per fully qualified GP than those in the least deprived areas—a disparity that has increased by 50% since October 2018 (10). This includes an appreciation that health outcomes are not distributed equally and that unrecognized structural obstacles such as poverty and housing instability or stigma condition patient experiences before ever arriving at the clinic. This also involves an understanding of responsibility not just to individual patients, but to an entire group a clinician has responsibility to, which includes those who are disengaged, under-served, voiceless, or marginalized.

Framed more broadly, population health is a vehicle for promoting health equity (11). It empowers GPs to recognize and respond to unjust disparities in care access, resource distribution, and clinical outcomes. In this way, population stewardship becomes not only a clinical responsibility but a moral one—rooted in a commitment to fairness for all.

It cannot be developed by mere exposure to data. Reports and measures might shed light upon trends, but they neither inspire reflection, nor convey context or meaning (12). To teach population health without an accompanying shift of thinking risks it becoming an exercise in technical compliance. To be fully active participants in dealing effectively with population health, GPs need to learn to discern stories behind statistics, systems behind symptoms, and accountability for patterns of care which they might not even fully grasp. This is not a skill—that is, it is not an ability which can very much be taught. It is an outlook, which must be developed explicitly through process and conversation and consistency over time.

## Continuity as the root of stewardship

Perhaps one of the most characteristic aspects of GP training in the United Kingdom is a concentration upon continuity of patient care. This approach is increasingly vital, as general practices in England delivered a record 31.9 million appointments in November 2023, averaging over a million appointments each working weekday (13). Trainees are most often assigned to a single general practice for a prolonged period of time, often between 6 and 18 months, during which time they form continuous relationships with a group of patients (14). This format not only develops clinical confidence and communication skills but has a central, though often unrecognized, function of instilling a population health mindset.

As time passes, and patients are seen regularly, patterns are uncovered that are not visible from individual consultations (15). The trainee could visit a patient whose asthma is not under control, who has missed two outpatient appointments, and do not know on their third visit that their condition is exacerbated by cold accommodation and by money worries. And it is longitudinally, through these regular contacts, that social explanations for medical conditions start to become evident. Continuity provides space for insight—not through structured teaching, but through intensive observation and incidental learning.

This type of learning creates a quiet but profound change. Instead of viewing patients as individual clinical episodes, trainees begin to feel like they belong to a larger group for whom they have a responsibility. They begin to observe who is no longer going, who is getting by quietly, and whose need is not being met. This attentiveness is crucial, as GPs in the most deprived areas are now responsible for caring for a staggering 2,450 patients each—over 300 more than their counterparts in more affluent areas (16). Crucially, it is not prompted by a dashboard, but by not seeing a familiar name on the clinic list, or an observation by a practice nurse who is aware of a patient's domestic situation.

The resulting sense of ownership is subtle. There is no official pronouncement that a trainee is responsible for a specified population. But the shape of continuity itself pushes them toward it. Through deepening relationships and familiarities, a professional sense of responsibility—not merely to attend when patients appear, but to think about those who don't–awakens. This is where population stewardship is seeded.

Patients, too, perceive when care moves beyond the individual consultation. Many value being known over time—not only for their symptoms but for their circumstances. When non-attenders are followed up, or when care anticipates unspoken needs, patients often describe a deeper sense of trust and belonging. Embedding patient perspectives into the evaluation of population health education may help trainees understand not only how to deliver care—but what that care feels like to those receiving it.

What continuity provides, therefore, is not just clinical richness, but ethical depth. Continuity brings to life the richness of real-world care and challenges trainees to start thinking about health not just individually, but about patterns, about systems, about responsibility to communities. And by doing that, it creates the mindset that population health requires.

## Integrating the ecosystem: interagency and community-based exposure

Alongside continuity of care, integration into the broader health and social services ecosystem is equally important in determining how GP trainees learn about population health. As of September 2024, general practice in England employed 148,853 full-time equivalent staff, with a total headcount of 197,683, underscoring the extensive network within which GPs operate (17).

Although the consultation room is an important setting for clinical learning, much that determines health happens outside of it. In the UK, trainees are regularly exposed to and engage in activities that bring them out of the consultation room and into this wider environment—sitting together for safeguarding meetings, working closely with community mental health teams, or referring patients through social prescribing routes. Such interagency encounters enable trainees to observe first-hand how housing, employment, education, and social support influence clinical outcomes (18).

This type of exposure creates a different sensibility—one where the GP is not just an individual actor, but one of a networked response to multifaceted human need (19). This subverts the linear model of diagnosis and treatment by insisting that each practitioner becomes an active participant with ambiguity, multiple parties, and aims that are likely to be negotiated rather than prescribed. Here, the GP's function becomes as much about coordination and advocacy as it is about diagnosis.

Effective coordination and advocacy depend on collaborative practice with other health professionals—such as nurses, pharmacists, physiotherapists, occupational therapists, mental health practitioners, and social workers. General practitioners must be trained not just in community awareness but in teamwork skills, interprofessional communication, and joint decision-making processes. Exposure to interprofessional learning fosters mutual understanding, reduces professional silos, and cultivates a culture of shared responsibility, which ultimately strengthens primary care as a cohesive and comprehensive frontline service. For example, trainees regularly engage in structured multidisciplinary team meetings involving nurses, pharmacists, social workers, and mental health professionals, addressing complex patient cases including chronic illness, polypharmacy, mental health issues, and social vulnerabilities. Trainees also undertake shadowing placements with community health workers or social prescribers, gaining direct experience of non-medical, community-based interventions that address social determinants of health.

Most importantly, these learning experiences are not merely information-based—they are relational and emotional. Trainees are exposed to professionals who function very differently from clinicians, and they learn that health enhancement is not merely a matter of medical intervention but of system navigation and collaboration. Gradually, it instills humility, flexibility, and a greater sense of responsibility. Exposure to diverse teams and patients also encourages trainees to reflect on how culture, language and migration status intersect with access and outcomes—prompting the development of cultural humility and more inclusive practice. Structured reflective practice sessions following these interprofessional engagements further embed trainees' learning by encouraging critical thinking on how collaborative practices directly influence patient outcomes and primary care effectiveness (20).

This exposure to non-medical professionals can encourage general practitioners to adopt a demedicalised approach to care, emphasizing social and community-based interventions rather than purely medical solutions. By broadening their understanding of health determinants and resources available in the community, practitioners are better positioned to reduce unnecessary medicalisation, potentially decreasing the tendency toward overprescribing medications, and prioritizing holistic, patient-centered strategies. Evidence indicates that such explicit interprofessional experiences significantly enhance trainee confidence, improve teamwork, reduce professional isolation, and support tangible improvements in patient-centered care delivery (20, 21).

While the exact mechanisms, like safeguarding boards or social prescribing initiatives, are specific to the UK, the principle itself is generalisable. However, providing trainees with structured and deliberate exposure to the social rather than clinical nature of care allows them to acquire the insight and understanding required to act effectively at population level. This type of ecosystem immersion is what brings about population health from an abstract to an experiential fact.

## Applied learning: the power of quality improvement and practice-level data

Though mindset and system exposure provide theoretical foundations for working in population health, developing the ability to take insight and apply it is critical. Quality improvement (QI) work provides one of the most practical mechanisms for this within the UK and is an obligatory part of GP training (22). This provides a structured but adaptable format through which trainees are able to get involved with data, analyse patterns, and drive improvement.

Trainees normally plan QI projects around local needs—for example, increasing take-up of cervical screening by women from underrepresented ethnic groups. In 2023–24, cervical screening coverage in England was 68.8%, highlighting the ongoing need for targeted interventions to improve uptake (18). They need to go beyond numbers. They need to communicate and get to know their colleagues, learn about local processes, investigate access barriers, and test the effect of small, focused interventions. QI then becomes more than an educational exercise—it is applied population health learning.

What is especially useful about these experiences is their proximity to real-world practice. Since trainees are working within the same GP practice over time, they are not applying theoretical changes within an abstract setting. They are working within a community that they are getting to know and often working for patients that they know individually. This integration of information and association brings relevance and purpose to the work.

However, QI's educational value is not intrinsic. If presented as box-ticking, it becomes quickly mechanical and disconnected from clinical practice and population health improvement (23). The difference lies in how the activity is contextualized: whether or not trainees are prompted to think through who is being omitted, why variation happens, and how their actions could alter outcomes. If explicitly connected to a deeper understanding of systems and an understanding of equity, QI doesn't just teach audit skills. QI teaches agency, strategic thinking, and a concrete sense of what it's like to act for a population.

Several practical interventions could support this development: (1) reflective exercises focused on missed or disengaged patients; (2) interagency shadowing logs capturing collaboration across sectors; (3) a population stewardship portfolio where trainees document health equity actions; and (4) faculty-led tutorials on interpreting panel-level variation. Embedding such practices turns abstract ideas into lived competencies (24).

## Identity formation: from clinician to community steward

As trainees progress through continuity-based placements, interact with interagency partners, and take part in applied quality improvement activities, an intangible outcome emerges that is not necessarily tested through curriculum goals or evaluated through portfolios: a transformation of professional identity. This evolution is not stated through curriculum aims or tested through portfolios but could be one of the most lasting outcomes of education for population health. Gradually, GP trainees tend to view themselves not merely as clinicians reacting to individual need, but as guardians of a community's health.

This identity formation is influenced by prolonged exposure to patterns of care, recurring gaps, and to patients whose lives go beyond episodic encounters (25, 26). A trainee GP who repeatedly encounters the same family—maybe through different generations—cannot help but adopt an extended sense of care. They soon begin anticipating what is required, get concerned about who has not returned, and are more proactive in advocating when systems fail. At such times, the clinician shifts from case managing to taking responsibility for populations.

Educational theory would inform us that identity is not created just by knowledge acquisition, but through engagement in significant practice. When trainees are given continuity, agency, and responsibility, then new professional norms are taken in by them. They understand that being a GP is not merely treating sickness, but about taking a longer view—a view over time, through lives, and through context and equity.

Importantly, no one dictates how this evolution happens; it emerges from one's association, reflection, and sense of proximity to others' lives. It is reinforced subtly: through a canceled appointment reverberating in one's mind, through a concern for safeguarding which spurs further investigation, or through a QI audit which brings to light an unseen group. These are the emotional and ethical scaffolds of population health practice. Through them, the GP not only becomes a provider, but an advocate for a population.

Measuring competencies in population health education typically involves direct assessment of knowledge application, reflective practice, and skills such as interpreting epidemiological data and evaluating intervention effectiveness. The use of structured competency frameworks, such as the Population Health Milestones introduced by the Accreditation Council for Graduate Medical Education (ACGME) in the US, allows educators to systematically track trainee progression (27, 28).

## Toward a global framework: cultivating the population health mindset

Though individual countries have different specific training structures for GP training, the overarching educational challenge is always the same one: how to equip future general practitioners not just to provide individual patient care, but also to take responsibility for a population's health (29). The mindset for such a role cannot be imparted through didactic content or technical aids alone. Rather, through deliberate, practice-based experiences, responsibility, reflection, and awareness of systems need to be embedded into the daily fabric of training.

Based on these themes that have been discussed throughout this article, a general framework for teaching population health to GP trainees can be outlined—one that is flexible across settings but rooted in three interdependent domains (see Table 1). The first is continuity and relationships, which generates longitudinal understanding and emotional responsibility. The second is exposure to community and system, which widens the clinician's lens to encompass social determinants and team-based care (30). The third is data and applied intervention, which allows trainees to analyse variation, test for improvement, and monitor outcomes. Combined, these domains teach more than knowledge; they define how trainees learn to perceive their role within their interaction with others.

**Table 1:** Embedding population health mindsets in GP training: a framework of experiential learning.

**Domain**	**Educational mechanism**	**What it teaches**	**Outcome on trainee thinking**
Continuity and relationships	Longitudinal placements, repeat consultations	Emotional responsibility, patient narratives	Ownership of community; noticing absence and patterns
System and community exposure	Interagency work, safeguarding meetings, social prescribing	Understanding social determinants, teamwork	GP as system navigator and advocate
Data and applied learning	Quality Improvement projects, practice-level audits	Variation analysis, equity in outcomes	Strategic action based on population insight
Reflective practice	Tutorials, narrative exercises, identity discussions	Meaning-making, moral reasoning	Awareness of professional role as population steward
Co-production with patients	Patient feedback, lived experience sessions	Patient-centered perspectives on equity and engagement	Practice grounded in real-world needs and community values

This model does not recommend a standard curriculum. Rather, it provides a series of guiding principles that can be applied across a variety of settings. While this paper draws from the UK model, similar imperatives are emerging worldwide. In the US, family medicine training incorporates resident-specific panel data and registries; in Australia, rural GP placements increasingly emphasize population outreach. Nordic countries embed health equity through early community-based rotations. These differences offer fertile ground for shared innovation and policy alignment (31). Furthermore, in US family medicine residency programs, trainees regularly engage in community-based projects, population health management using electronic health records, and structured collaborations with public health departments. These experiences are designed to embed population health competencies through experiential learning (32, 33).

Whether you are working in a rural clinic that has scant infrastructure or an urban health system that has sophisticated tools for data capture and analysis, the intent is consistent: to equip trainees to view populations, not patients (34). Increasingly, education leaders are recognizing the value of co-producing population health curricula with patients, carers, and community groups—ensuring that training remains grounded in lived realities and responsive to diverse needs. This capacity is what will characterize the future generation of general practitioners. Several medical schools have explicitly integrated population health competencies into their curricula, demonstrating measurable outcomes in trainee competencies and community engagement. These structured curricula can serve as valuable models internationally (35, 36).

## Conclusion—what kind of GPs are we training?

As health system demands shift, so must general practitioner education and continuing professional development. Population health is not a new toolkit or an administrative overlay, but a new way of perceiving, thinking, and acting within clinical practice. To educate GPs to address community and individual need, we need to look beyond teaching about interpreting data or coordinating care. We need to generate a learning environment that instills a deep sense of ownership, a curiosity about pattern and process, and a commitment to equity.

There is also a need to recognize the emotional and cognitive burden that population responsibility may bring. Without appropriate mentorship and team support, the shift from individual to population-level care can risk moral fatigue or overwhelm. Training must cultivate not only insight and agency, but also resilience and realistic expectations—helping future GPs navigate the space between idealism and sustainability.

This cannot be done by teaching alone. Continuity, immersion, and reflection are needed—conditions through which professional identity can be built, based upon stewardship and not merely upon service provision. Whether through dashboards or through years of association, it is the same goal: to educate GPs who understand that accountability does not stop when the consultation is over, but starts from and continues through to the population whose health they are managing.
